# Comparison of microendoscopic selective laminectomy versus conventional laminoplasty in patients with degenerative cervcical myelopathy: a minimum 2-year follow-up study

**DOI:** 10.1186/s12891-019-2884-6

**Published:** 2019-10-25

**Authors:** Yasushi Oshima, So Kato, Toru Doi, Yoshitaka Matsubayashi, Yuki Taniguchi, Sakae Tanaka

**Affiliations:** Department of Orthopaedic Surgery, The University of Tokyo, 7-3-1 Hongo, Bunkyo-ku, Tokyo, 113-8655 Japan

**Keywords:** Posterior surgery, Spondylosis, Ossification of the posterior longitudinal ligament, Minimally invasive surgery

## Abstract

**Background:**

Although microendoscopic partial laminectomy for patients with degenerative cervical myelopathy (DCM) has been reported and demonstrated good results, a detailed comparison of its mid-term surgical results with those of laminoplasty (LP) has not been reported. The aim of this study was to compare the surgical outcomes, complications, and imaging parameters of cervical microendoscopic interlaminar decompression (CMID) via a midline approach versus conventional laminoplasty, with a minimum follow-up period of 2 years.

**Methods:**

Two hundred and fifty-four patients who underwent either LP or CMID for DCM between May 2008 and April 2015 were enrolled. All patients routinely underwent LP (C3–6 or C3–7) before December 2011, whereas CMID was performed at the one or two affected level(s) only in patients with single- or two-level spinal cord compression after 2012.

*Surgical procedure (CMID)*: For single-level patients (e.g., C5–6), partial laminectomy of C5 and C6 was performed under a microendoscope. For two-level patients (e.g., C5–6-7), decompression was completed by performing a C6 laminectomy.

We compared surgical outcomes and radiographic parameters between the CMID and LP groups.

**Results:**

Of the 232 patients followed up for > 2 years, 87 patients with single- or two-level spinal cord compression, 46 that underwent CMID, and 41 that underwent LP were identified. There were no differences in the baseline demographic data of the patients between the groups. CMID showed better outcomes in terms of postoperative axial pain and quality of life, although both procedures showed good neurological improvement. Two and one patient complained of C5 palsy and hematoma, respectively, only in the LP group. The postoperative range of motion was worse and the degree of postoperative posterior spinal cord shift was larger in the LP group.

**Conclusion:**

Selective decompression by CMID demonstrated surgical outcomes equivalent to those of conventional LP, which raises a question regarding the requirement of extensive posterior spinal cord shift in such patients. Although the indications of CMID are limited and comparison with anterior surgery is mandatory, it can be a minimally invasive procedure for DCM.

## Background

Patients with degenerative cervical myelopathy (DCM) usually have numbness and dexterity of the upper extremities, gait disturbances, and bowel and bladder dysfunction. Because these symptoms can gradually deteriorate, surgical intervention is indicated in patients with moderate-to-severe compression of the spinal cord or who show a progressive course [6].

Laminoplasty (LP) has been shown to be an effective treatment for patients with DCM [2] [24]. The main concept of this procedure has been a posterior shift of the spinal cord, and the surgical levels basically included vertebrae from C2 or C3 to C6 or C7, regardless of the number of levels with spinal cord compression [26]. This is reasonable because in a posterior approach, posterior factors (lamina, facet joints, and yellow ligament) can only be treated and anterior factors (disc bulging and osteophyte formation) cannot be removed.

On the other hand, there have been many reports on surgery-related complications following LP such as postoperative axial neck pain [12] [15], kyphosis [23], reduced range of motion (ROM) [3] [25], and the so-called C5 palsy [3]. To overcome these problems, several less invasive techniques such as muscle-preserving selective laminectomy [25] and microendoscopic laminectomy [31] [30] [17] [5] [21], that do not require extensive spinal cord shift, have been reported and demonstrated good results; however, a comparison of its mid-term surgical results with those of LP remain uncertain. The aim of this study was to compare the surgical outcomes, complications, and imaging parameters of cervical microendoscopic interlaminar decompression (CMID) via a midline approach versus conventional laminoplasty, with a minimum follow-up period of 2 years.

## Methods

This was a comparative historical analysis of 254 prospectively enrolled patients who underwent posterior decompression surgery without fusion for cervical spondylotic myelopathy (CSM) or ossification of the posterior longitudinal ligament (OPLL) (occupying ratio < 40%) between May 2008 and April 2015. Patients with an anterior slip (> 3 mm), kyphosis (> 10°), disc herniation, OPLL with an occupying ratio ≥ 40%, rheumatoid arthritis, and a history of trauma or past surgery were not included. All patients routinely underwent LP (C3–6 or C3–7) based on the concept of posterior spinal cord shift before December 2011, whereas CMID was performed at the one or two affected level(s) only in patients with single- or two-level spinal cord compression after January 2012. The level(s) that was considered responsible for the myelopathic symptom was determined by neurological examinations by at least two spine surgeons as well as by complete obstruction of the subarachnoid space and spinal cord compression on preoperative T2-weighted magnetic resonance imaging (MRI). No patients that met the criteria underwent anterior surgery or posterior fusion surgery during this period.

### Surgical procedure (CMID)

**Fig. 1:**
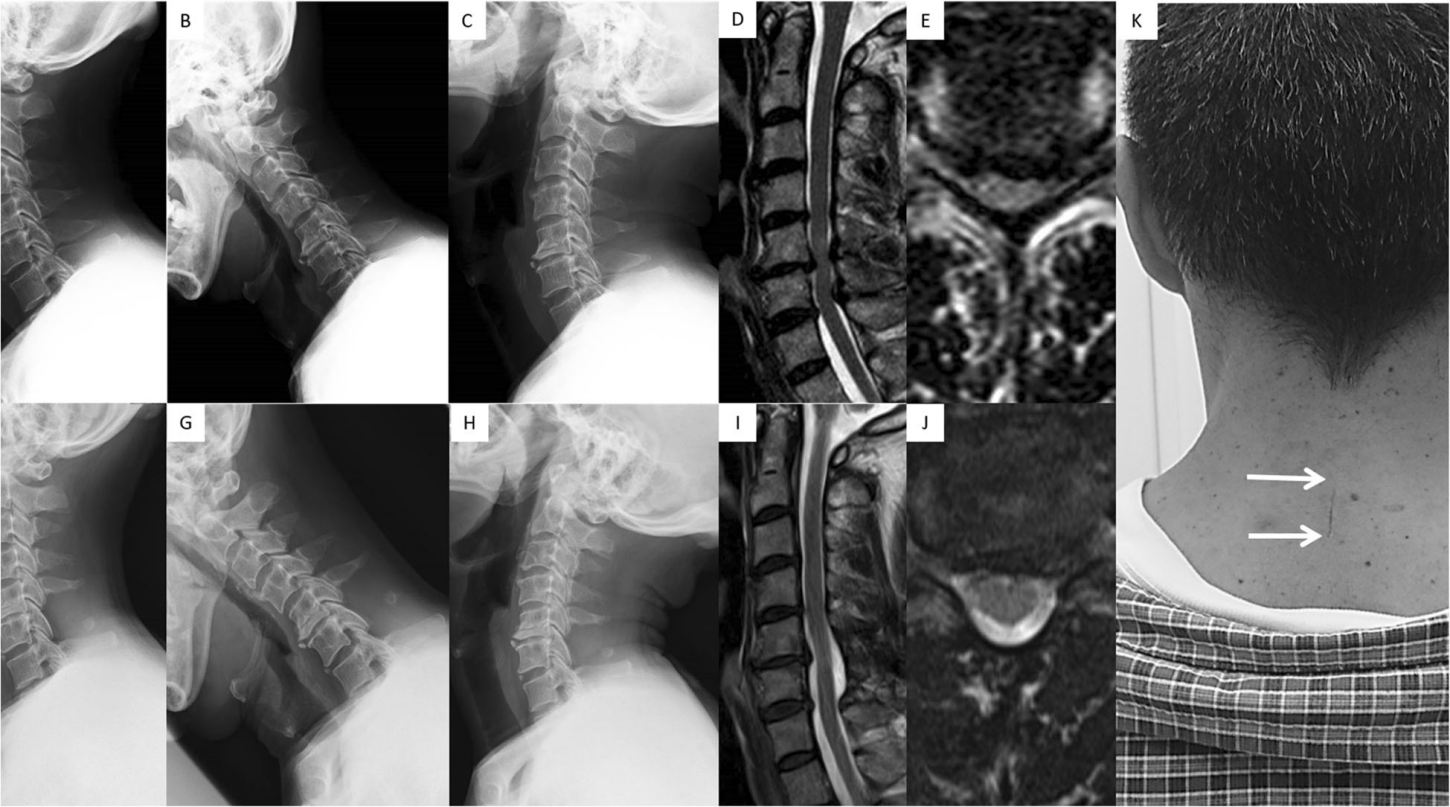
A 58-year-old man presented with two-level spinal cord compression at C5/6 and C6/7 and underwent cervical microendoscopic interlaminar decompression (CMID) from C5 to C7; i.e., laminectomy of C6 and partial laminectomy of C5 and C7 after splitting the C6 spinous process. **a, b, c** Preoperative lateral X-ray in neutral (**a**), flexion (**b**), and extension (**c**) positions. **d, e** Preoperative sagittal (**d**) and axial (**e**) images on T2-weighted magnetic resonance imaging (MRI). **f, g, h** Postoperative (at 28 months) lateral X-ray in neutral (**f**), flexion (**g**), and extension (**h**) positions. **i, j** Postoperative sagittal (**i**) and axial (**j**) images on T2-weighted MRI. **k** Clinical photograph showing the operative scar (arrows). Pre−/postoperative C2/7 Cobb angles, range of motion, spinal cord diameter, and degree of posterior spinal cord shifting were 10/10 degrees, 50/48 degrees, 3.0/5.2 mm, and 2.6 mm, respectively. The pre- and postoperative Japanese Orthopedic Association (JOA) scores were 10 and 16

CMID was performed as previously described [21]. For single-level cases (e.g., C5–6), an approximately 2-cm midline skin incision was made under fluoroscopic guidance at the spinal level to be decompressed (C5–C6) (Fig. 1). The nuchal ligament was longitudinally cut, and the tips of the spinous processes (C5 and C6) were exposed. A 16-mm tubular retractor was inserted onto the tips of the C5 and C6 spinous processes. The set of the METRx endoscopic system (Medtronic Sofamor Danek, Memphis, TN, USA) was used for the microendoscopic procedure. The tips of the spinous processes were occasionally drilled to make the retractor stable. Spinous processes were partially cut to make a working space for the decompression procedure when the interspinous space was narrow. The interspinales muscles were spread bluntly. Deep attachment of the semispinalis cervicis and multifidus muscles was partly coagulated and dissected. Subsequently, a dome-like laminectomy of C5 and partial laminectomy of the upper part of C6 together with a flavectomy were performed, and single-level decompression was completed. After the decompression, the working channel was carefully removed and a drain was placed. The nuchal ligament and skin were closed by using standard techniques. For two-level cases (e.g., C5–6 and C6–7), the C6 spinal process was first split with a burr and divided at its base. A C5 and C7 dome-like laminectomy and C6 laminectomy with an approximately 16-mm width were performed. After surgery, the drain was taken out after 2 days. Patients did not wear a neck collar.

### Surgical procedure (LP)

We performed double-door LP as previously described [24]. Surgical levels were C3 to C6 (or C7 when C6/7 was involved) in all cases. C2 was also opened when necessary but was not involved in any of the cases of with single- or two-level cord compression in this series. Therefore, the attachment of semispinalis muscles to C2 was preserved in all cases with single- or two-level cord compression. After cutting the nuchal ligament, cervical laminae were exposed laterally to the medial aspect of the facet joints, and the interspinous ligaments were removed. The spinous processes were split sagittally. After bilateral gutters for the hinges were carefully made at the transitional area between the facet joint and laminae, spinal canal enlargement was achieved by bilateral opening of the laminae. Hydroxyapatite spacers (Boneceram; Olympus Terumo Biomaterials Corp., Tokyo, Japan) were placed between the opening laminae and fixed with nonabsorbable sutures. The drain was removed 2 days after surgery. The patients wore a soft cervical orthosis for approximately 1 to 2 weeks, depending on the degree of postoperative neck pain.

### Radiographic evaluation and outcome measurements

We compared surgical outcomes and radiographic parameters between the CMID and LP groups in patients having a single- or two-level spinal cord compression. Regarding the radiographic parameters, the C2–7 Cobb angles, ROM, C2–7 sagittal vertical axis (C2–7 SVA), and segmental lordotic angle at the level(s) of spinal cord compression were measured. In the MRI evaluations, the spinal cord diameter and the degree of posterior shift of the spinal cord were measured. The distance from the posterior wall of the vertebral body to the center of the spinal cord at the level of maximum spinal cord compression was measured pre- and postoperatively, and the difference was defined as the degree of posterior spinal cord shifting [9]. The primary outcomes were the Japanese Orthopedic Association score (JOA score), the Neck Disability Index, the Short Form 36 (SF-36), the EuroQol 5 Dimension, and the numerical rating scale score (0–10) for arm, neck, and scapular pain. Satisfaction was evaluated by using a seven-point scale as follows: very satisfied, satisfied, slightly satisfied, neither satisfied nor dissatisfied, slightly dissatisfied, dissatisfied, and very dissatisfied. Patients were divided into two groups: satisfied (very satisfied, satisfied, slightly satisfied) and dissatisfied (neither satisfied nor dissatisfied, slightly dissatisfied, dissatisfied, very dissatisfied) [19].

### Statistical analysis

SPSS v.18 software (SPSS, Chicago, IL, USA) was used to perform the Mann–Whitney U test and chi-square test. All *p*-values were two-sided, and a *P* value of <.05 was considered as indicative of statistical significance.

## Results

**Fig. 2:**
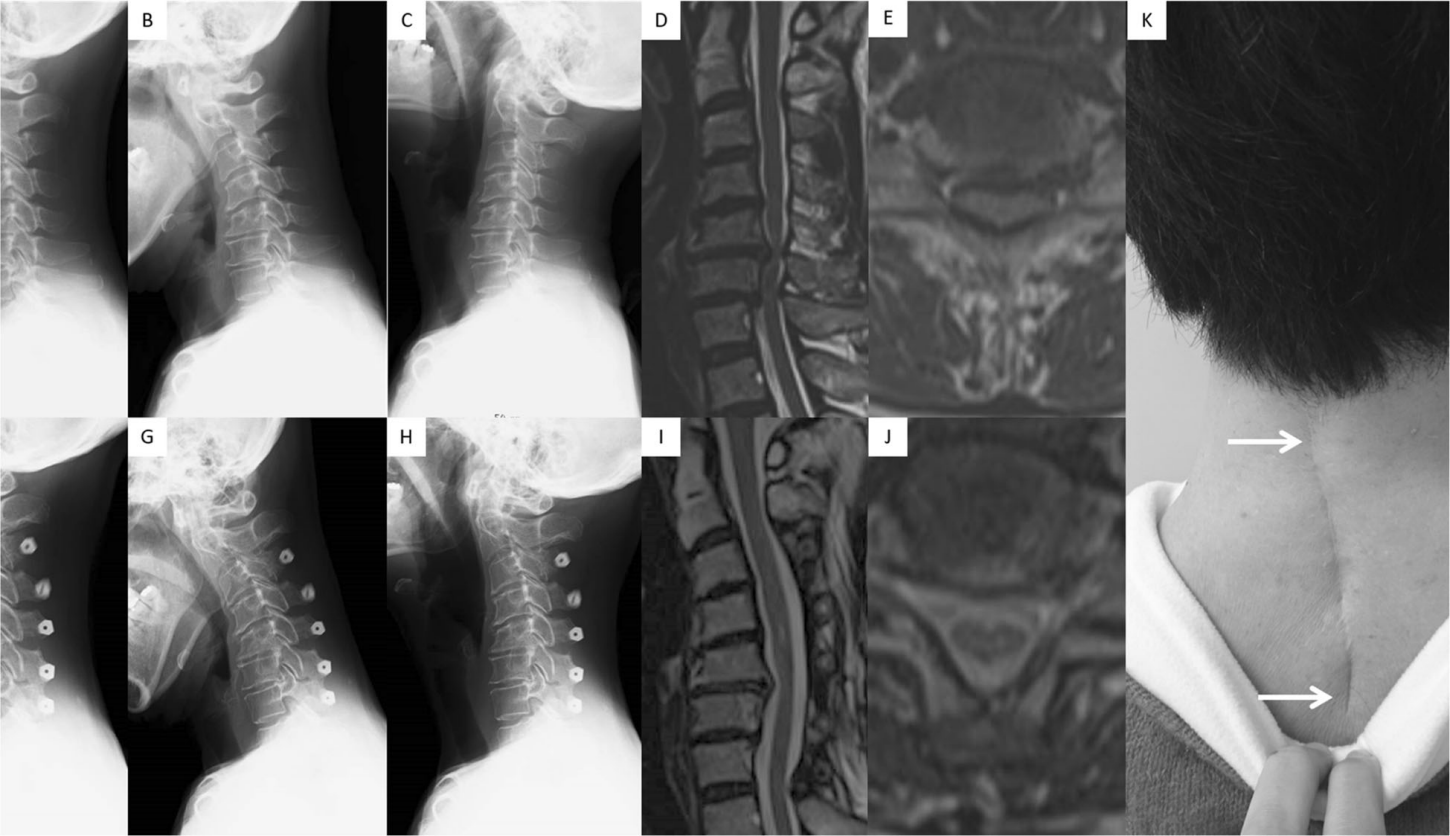
A 62-year-old woman presented with two-level spinal cord compression at C5/6 and C6/7 and underwent cervical laminoplasty (LP) from C3 to C7. **a, b, c** Preoperative lateral X-ray in neutral (**a**), flexion (**b**), and extension (**c**) positions. d, e Preoperative sagittal (**d**) and axial (**e**) images on T2-weighted magnetic resonance imaging (MRI). **f, g,h** Postoperative (at 28 months) lateral X-ray in neutral (**f**), flexion (**g**), and extension (**h**) positions. i, j Postoperative sagittal (**i**) and axial (**j**) images on T2-weighted MRI. **K,** Clinical photograph showing the operative scar (arrows). Pre−/postoperative C2/7 Cobb angles, range of motion, spinal cord diameter, and degree of posterior spinal cord shifting were 0/− 10 degrees, 56/19 degrees, 3.6/5.7 mm, and 3.8 mm, respectively. The pre- and postoperative Japanese Orthopedic Association (JOA) scores were 10 and 13. This patient showed progressive kyphosis postoperatively and complained of neck pain (numerical rating scale score of 7)

Two hundred and thirty-two (91%) patients completed the 2-year follow-up, and 87 (37%) had single- or two-level spinal cord compression. Of these, 41 patients underwent LP before December 2011, whereas 46 underwent CMID after January 2012. There was no significant difference in the baseline demographic data of the patients between the two groups (CMID vs. LP, respectively, all): average age, 63.4 vs. 64.5 years; male/female, 32/14 vs. 23/18; follow-up period, 27.8 vs. 27.3 months; and CSM/OPLL, 35/11 vs. 30/11 (Table 1). Moreover, there were no significant differences in the preoperative outcomes and radiographic parameters (Table 2, Table 3). The mean estimated intraoperative blood loss was 18 ml (range, 0–100 ml) in the CMID group, whereas 93 ml (range, 0–430 ml) in the LP group (*P* < 0.01). Among the postoperative outcomes, neck, arm, and scapular pain, physical function, and general health of the SF-36 were significantly better in the CMID group than in the LP group, whereas the JOA score was not significantly different (Table 2). The proportion of patients who were satisfied with the treatment was significantly higher in the CMID patients (82% in CMID versus 56% in LP, *P* = .007). The postoperative ROM was worse, whereas the degree of postoperative posterior spinal cord shift on MRI was larger in the LP group (Table 3). Two patients and one patient complained of C5 palsy and hematoma, respectively, in the LP group, whereas there were no complaints in the CMID group. Illustrative cases of each procedure are demonstrated in Fig. 1 and Fig. 2.

**Table 1:** Demographic data of patients

	CMID (*n* = 46)	LP (*n* = 41)	*P*-value
Age (yr)	63.4 (SD, 14.2)	64.5 (SD, 10.2)	0.70
Sex (M/F)	32/14	23/18	0.19
Follow-up (mo)	27.8 (SD, 6.0)	27.3 (SD, 5.4)	0.65
BMI (kg/m^2^)	23.0 (SD, 2.7)	23.5 (SD, 3.4)	0.43
CSM/OPLL	35/11	30/11	0.76
Spinal cord compression			
One-level/Two-level	18/28	13/28	0.47

*Continuous variables were compared using the Mann–Whitney U test; categorical data were analyzed using the chi-square test. Significant values are p < 0.05*

**Table 2:** Comparison of preoperative and postoperative outcomes between CMID and LP

		CMID		LP		*P*-value
		average	SD	average	SD	
Numerical rating scale						
Neck	*pre*	3.3	2.7	3.7	3.2	0.52
	*post*	1.3	1.8	3.0	2.4	0.00
Arms	*pre*	3.8	2.8	4.4	3.0	0.35
	*post*	1.7	2.1	3.4	2.5	0.00
Scapular lesion	*pre*	2.0	2.6	2.2	2.6	0.64
	*post*	1.1	1.6	2.7	2.5	0.00
NDI	*pre*	34.0	15.5	35.2	18.9	0.74
	*post*	21.9	15.1	28.1	16.2	0.08
EQ 5D	*pre*	0.58	0.11	0.57	0.17	0.94
	*post*	0.73	0.17	0.66	0.19	0.07
SF-36						
Physical Functioning	*pre*	29.6	22.9	27.6	16.4	0.66
	*post*	41.5	18.4	31.5	16.7	0.01
Role Physical	*pre*	29.7	18.0	26.5	15.0	0.40
	*post*	39.3	13.9	34.1	16.2	0.13
Bodily Pain	*pre*	37.2	0.7	36.1	9.8	0.65
	*post*	44.6	10.1	40.8	11.3	0.11
General Health	*pre*	44.0	9.3	41.8	9.4	0.30
	*post*	46.2	9.0	41.2	12.3	0.04
Vitality	*pre*	42.0	12.8	41.9	12.9	0.95
	*post*	45.1	12.9	44.1	11.6	0.70
Social Functioning	*pre*	37.1	16.5	34.4	15.7	0.46
	*post*	43.1	12.7	40.4	14.3	0.37
Role Emotional	*pre*	38.8	19.2	32.3	17.1	0.12
	*post*	43.7	15.5	38.9	15.2	0.16
Mental Health	*pre*	44.7	12.9	40.8	15.4	0.23
	*post*	48.2	10.7	47.6	11.4	0.81
JOA score	*pre*	10.5	2.9	10.5	2.2	0.92
	*post*	13.2	2.7	13.3	2.1	0.84
	*recovery rate*	45.7	28.0	43.7	29.9	0.75

*Continuous variables were compared using the Mann–Whitney U test. Significant values are p < 0.05*

**Table 3:** Comparison of pre- and postoperative imaging parameters between CMID and LP

		CMID		LP		*P*-value
		average	SD	average	SD	
C27 Cobb (degrees)	pre	11.0	8.0	9.5	9.3	0.45
	post	10.7	8.7	11.5	12.4	0.76
C27 ROM (degrees)	pre	41.8	10.8	44.3	14.5	0.39
	post	42.1	10.6	28.2	12.4	0.00
C27 SVA (mm)	pre	1.9	1.4	2.0	1.2	0.76
	post	1.6	1.2	2.1	1.5	0.16
Segmental lordotic angle (degrees)	pre	5.9	5.7	4.4	6.4	0.30
	post	5.1	5.9	3.1	6.9	0.17
Spinal cord diameter (mm)	pre	4.2	0.9	4.2	0.7	0.76
	post	5.7	1.1	5.9	0.7	0.44
Posterior spinal cord shift (mm)		1.9	1.1	2.9	1.4	0.00

*Continuous variables were compared using Mann–Whitney U test. Significant values are p < 0.05*

## Discussion

We sought to assess the advantages and disadvantages of CMID surgery versus conventional LP, and two important findings were made: First, CMID showed better surgical outcomes than LP in terms of postoperative pain and quality of life, although both procedures showed good neurological improvement as evidenced by the JOA score recovery rate. Second, the posterior shift of the spinal cord was significantly smaller in the microendoscopic procedure.

As stated in the introduction, cervical LP is an established procedure in patients with DCM and is particularly effective in those with multilevel spinal cord compression and lordotic alignment. Cervical ROM is decreased but still preserved after LP. On the other hand, postoperative axial neck pain and postoperative reduced ROM or kyphosis have been reported as disadvantages of this procedure. Because less invasive and muscle-preserving techniques of LP have shown better results in terms of postoperative neck pain in the literature [11], it was reasonable that patients following CMID complained of less pain postoperatively than did those following LP. We speculate that this is mainly because of the minimalized damage to the neck structure. Furthermore, limited ROM was not demonstrated in patients with CMID, which might be another reason for the absence of postoperative axial pain in those undergoing CMID. It was surprising that postoperative cervical alignment was maintained in patients with LP. We speculate that this is because the attachment of semispinalis muscles to the C2 spinal process was preserved in the cases involved [28]. Although the reason why patients after CMID showed less pain in the arm remains uncertain, patients complaining of postoperative axial neck pain tend to have a lower mental state and are susceptible to pain. We speculate that such patients showed increased pain in the arm as well as in the neck after LP, which was not very common in the CMID group.

In this study, posterior shift of the spinal cord was significantly smaller in patients following CMID in which partial laminectomy with a width of approximately 16 mm and removal of yellow ligament had been performed. On the other hand, posterior shift of the spinal cord has been considered to be the main concept of LP [26] [24]. Because this study did not include such patients with kyphosis or massive anterior factors, the effects of posterior decompression were similar regardless of the degree of posterior shifting of the spinal cord. We assume that the effects of posterior decompression do not significantly differ between CMID and LP when patients have good cervical alignment. Given that too much posterior shifting of the cord can cause irritation of the nerve root and C5 palsy [13], we question the need for posterior shift of the spinal cord in such cases and assume that patients with good cervical alignment will be an indication for this method.

However, it seems apparent that the indications for CMID should be more limited than those for LP. Regarding the indications of LP, it has been reported that patients with kyphosis > 13° [27], sigmoid alignment [29], anterior slip [20], or large OPLL [8] should be contraindicated. This is the limitation of posterior decompression by LP, and such patients should undergo anterior surgery or posterior decompression with fusion. Because CMID has a small effect on the posterior shift of the spinal cord, the indication should be more limited. Indeed, patients with kyphosis > 10°, an anterior slip ≥3 mm, or an OPLL with an occupying ratio of ≥40% did not undergo CMID; we do not recommend this method to be applied to such patients.

One more concern in selective decompression is stenosis at other levels in the future, particularly in patients with developmental canal stenosis. The occurrence of adjacent segment pathology after anterior fusion surgery is well known [10, 16]. Although the incidence of adjacent segment pathology would be lower after posterior decompression surgery without fusion [16], the possibility of revision surgery should be communicated to the patients before surgery, particularly those with developmental canal stenosis. Furthermore, when there is a discrepancy between neurological level diagnosis and MRI findings, myelography will be effective to decide the surgical levels to be decompressed. A much longer follow-up study of over ≥10 years will be necessary to clarify this matter.

The posterior approach we used in the microendoscopic surgery was through a midline intermuscular approach after cutting the nuchal ligament, which is different from that through a paramedian intramuscular approach, more generally used. Microendoscopic decompression surgery was originally reported as a treatment for lumbar disc herniation [7] [22], but is now applied to lumbar spinal canal stenosis and cervical spondylosis [1] [4] [17]. The original approach is unilateral, with a skin incision placed 10 mm off the midline via an intramuscular approach. Ipsilateral laminectomy and laminectomy of the contralateral side is performed. Although this approach is effective in patients with lumbar spinal canal stenosis, we consider that the maneuver is often dangerous in cervical spine cases because surgeons could fall into retracting and compressing the spinal cord in an attempt to view and decompress the contralateral side. In addition, asymmetrical decompression can cause irritation or tethering of the nerve root, which leads to radicular pain or C5 palsy [17] [13] [32]. Moreover, a paramedian intramuscular approach off the midline causes muscle trauma and bleeding, which can lead to a postoperative hematoma and palsy [14, 18]. Therefore, we have used an intermuscular approach through a midline when using a microendoscopic system. We speculate that this is why no patients complained of C5 palsy or hematoma in the present series.

Although we used a microendoscopic system with a 16 mm-tubular retractor, selective laminectomy under a microscopic field, a slightly longer skin incision, and muscle exploration would have given similar surgical results. We encourage surgeons who are not accustomed to using a microendoscopic system to try selective laminectomy under a microscope. Indeed, there is a steep learning curve in spinal microendoscopic surgery. What we wish to emphasize is not the best uses of a microendoscopic system, but rather the concept of posterior decompression by selective laminectomy through a median approach.

There were several limitations in this study. First, this was not a randomized controlled study, although the patients were historically controlled. Second, the long-term results of the selective microendoscopic procedure remain uncertain. Restenosis or stenosis at another level may occur in the future. Third, comparison with anterior surgery should be performed because many doctors will prefer an anterior approach in patients with single- or two-level spinal cord compression. Because adjacent segment pathology owing to fixation surgery will not occur after CMID, patients with developmental canal stenosis will have a good indication for CMID. Fourth, this study included patients with OPLL as well as those with CSM, although we understand that the etiologies are different. However, it has been demonstrated that neurological improvement did not differ between patients with CSM and smaller OPLL [24]. It is no doubt that CMID has a good indication when spinal cord compression is caused by a pincer mechanism by spondylosis or small OPLL at a single level, although total laminectomy by CMID will cover continuous OPLL to some extent. Nevertheless, we emphasize that the indication of CMID should be more limited as we stated above. Finally, we did not check the difference at an early stage after surgery, because this study aimed to investigate mid-term to long-term surgical results. CMID might have shown better results in terms of perioperative pain, length of hospital stay, or time to return to work. Further investigation will provide a better understanding of these problems.

## Conclusion

Selective decompression by CMID demonstrated surgical outcomes equivalent to those of conventional LP of C3–6 or C3–7, which raises a question as to the need for posterior spinal cord shift in such patients. Furthermore, the microendoscopic procedure via a midline intermuscular approach minimized blood loss and muscle trauma, which resulted in no hematoma and less postoperative pain. Although the indications of CMID are limited and comparison with anterior surgery is mandatory, it can be a minimally invasive procedure for DCM. 

## Abbreviations

CMID: Cervical microendoscopic interlaminar decompression
CSM: Cervical spondylotic myelopathy
DCM: Degenerative cervical myelopathy
LP: Laminoplasty
OPLL: Ossification of the longitudinal ligament
